# Female “Paradox” in Atrial Fibrillation—Role of Left Truncation Due to Competing Risks

**DOI:** 10.3390/life13051132

**Published:** 2023-05-05

**Authors:** Tomoki Nakamizo, Munechika Misumi, Tetsuya Takahashi, Satoshi Kurisu, Masayasu Matsumoto, Akira Tsujino

**Affiliations:** 1 Department of Clinical Studies, Radiation Effects Research Foundation (RERF), Nagasaki 850-0013, Japan; 2 Department of Statistics, Radiation Effects Research Foundation (RERF), Hiroshima 732-0815, Japan; 3 Faculty of Rehabilitation, Hiroshima International University, Hiroshima 739-2695, Japan; 4 Department of Clinical Studies, Radiation Effects Research Foundation (RERF), Hiroshima 732-0815, Japan; 5 Iseikai Hospital, Osaka 533-0022, Japan; 6 Department of Neurology and Strokology, Nagasaki University Hospital, Nagasaki 852-8501, Japan

**Keywords:** atrial fibrillation, stroke risk, risk factors, sex difference, race/ethnicity difference, survival analysis, competing risks, frailty models, left truncation, collider bias

## Abstract

Female sex in patients with atrial fibrillation (AF) is a controversial and paradoxical risk factor for stroke—controversial because it increases the risk of stroke only among older women of some ethnicities and paradoxical because it appears to contradict male predominance in cardiovascular diseases. However, the underlying mechanism remains unclear. We conducted simulations to examine the hypothesis that this sex difference is generated non-causally through left truncation due to competing risks (CR) such as coronary artery diseases, which occur more frequently among men than among women and share common unobserved causes with stroke. We modeled the hazards of stroke and CR with correlated heterogeneous risk. We assumed that some people died of CR before AF diagnosis and calculated the hazard ratio of female sex in the left-truncated AF population. In this situation, female sex became a risk factor for stroke in the absence of causal roles. The hazard ratio was attenuated in young populations without left truncation and in populations with low CR and high stroke incidence, which is consistent with real-world observations. This study demonstrated that spurious risk factors can be identified through left truncation due to correlated CR. Female sex in patients with AF may be a paradoxical risk factor for stroke.

## 1. Introduction

The prevalence of atrial fibrillation (AF) increases rapidly with age [1,2,3], imposing a heavy burden of AF-related stroke among older people worldwide [1]. The risk of AF-related stroke can be reduced by oral anticoagulation therapy [3]. In the clinical setting, physicians decide on anticoagulation therapy based on the predicted stroke risk in their patients. Risk prediction is usually made by CHA_2_DS_2_-VASc scoring [4], which incorporates known risk factors, such as hypertension and prior stroke. A score of one or two points out of a total of eight is considered the threshold [3]. 

Female sex is given one point in the CHA_2_DS_2_-VASc [4], and the latest meta-analysis shows that its integrated hazard ratio (HR) for stroke is 1.24 [5]. However, it is a controversial risk factor because it predicts stroke inconsistently, as it does so among older women but not among younger women [5,6,7,8,9,10]. In addition, female sex predicts stroke in European populations but not in Asian populations [5,11], especially in populations with high stroke and low coronary artery disease (CAD) incidence, such as Taiwan [12], Hong Kong [13], Korea [14], and Japan [15]. These inconsistencies have led to the latest recommendation to avoid anticoagulation in patients with AF whose risk factor is female sex alone [3]. In addition, female sex as a risk factor appears to contradict the fact that cardiovascular diseases, such as CAD and even stroke in general, occur more frequently among men than women [5,16,17,18,19,20]. Therefore, it is paradoxical that sex acts in opposite directions only in AF-related stroke. This paradox also occurs in patients with AF: stroke occurs more frequently among women, but CAD, cardiovascular deaths, and overall deaths occur more frequently among men [5,21]. Although researchers have speculated about potential mechanisms, including reproductive hormones, differences in clinical practice, and residual confounding [10,22,23,24], it is unclear why female sex predicts stroke in patients with AF, why it does so preferentially among older European women, and where the apparent paradox comes from. 

Disease risk factors are usually identified through survival analysis, which assumes non-informative censoring and non-informative left truncation (LT), i.e., individuals drop out “independently” of the outcome during and before the follow-up. When there are competing risks (CR) and unobserved heterogeneity correlated with the outcome of interest, it may lead to informative censoring, which could generate a spurious association between the outcome and the variables associated with CR [25], a phenomenon called “false protectivity” under some circumstances [26]. 

Based on this phenomenon, the age/ethnicity dependency and the apparently opposite prediction described above, as well as the fact that AF is prevalent among older people, we hypothesize that female sex in AF is non-causally associated with stroke because of informative LT caused by premature deaths from CR, such as CAD, which share common, unobserved causes with stroke [4,27,28,29]. Other male-predominant diseases that share risks with stroke, including cancer [30], hepatic disease [31], and respiratory disease [32,33], may also be included in the CR. This study aimed to examine this hypothesis through simulations.

## 2. Methods

### 2.1. Population

**Figure 1:**
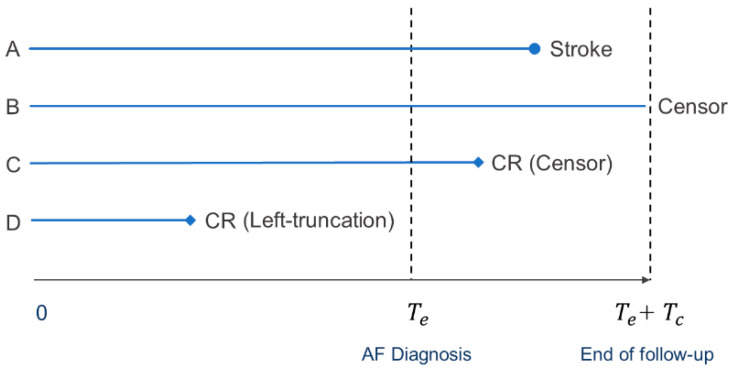
Follow-up of the study population. Four possible trajectories are shown: (A) stroke in AF, (B) no stroke in AF, (C) censor due to CR, and (D) left truncation due to CR.

Suppose a hypothetical population is composed of *N* same-aged persons of each sex, free from AF, who will eventually develop AF. We were interested in incident stroke after the diagnosis of AF. We assumed that the major CR is a cardiovascular death, mainly associated with CAD. However, CR can encompass other diseases with a correlated risk with stroke and with higher and earlier mortality in men than women. Hence, we define CAD in our model as a collective representation of all potential CR. We defined time 0 as the age at which CAD began to develop in this population (typically, 40 years). For simplicity, we assumed that all individuals were diagnosed with AF at time $T_{e}$, and defined the cohort of patients with AF at that time. The AF cohort was left-truncated; it consisted only of persons who were alive at the time of AF diagnosis and free from CAD. We followed up the cohort for subsequent strokes over a prespecified period $T_{c}$ (Figure 1). Individuals were censored if they did not develop a stroke before the end of the study or if they developed CR before stroke during the follow-up period. 

### 2.2. Model

**Figure 2:**
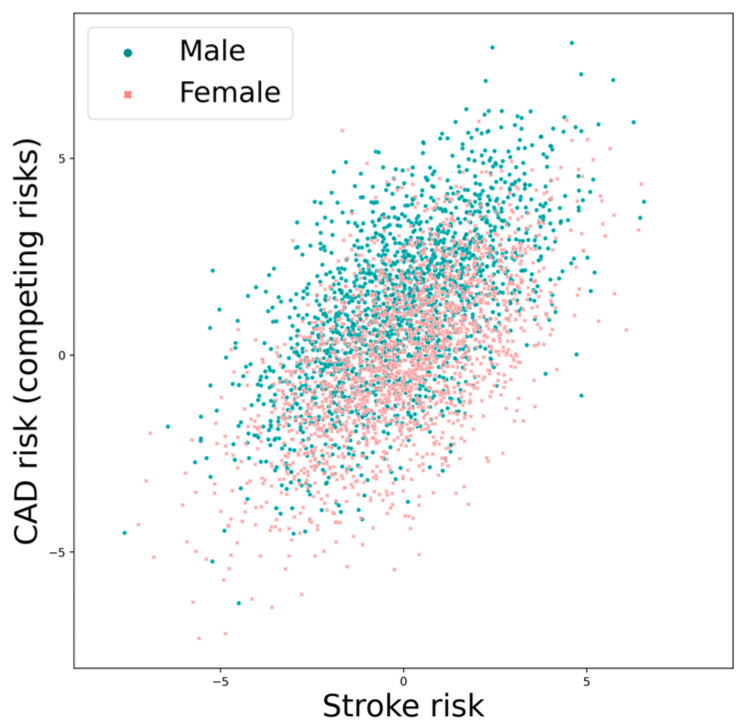
Individual risks (log hazard) of stoke and CAD (CR) in a simulated population. The risks are correlated among individuals, and men have a systematically higher risk for CAD than women. Parameters were set to $\sigma_{1}=\sigma_{2}=2,\;\rho =0.6$, and $\beta_{2}=1.2$.

We modeled stroke and CAD risks such that they are heterogenous and correlated among individuals and that men have a higher risk for CAD than women (Figure 2). We assumed constant hazards for simplicity. Let $\lambda_{1}\left[i\right]$ and $\lambda_{2}\left[i\right]$ be the log hazards of an *i*th individual for AF-related stroke and CAD (representative of all CR combined), respectively. We model

(1) $$\lambda_{1}\left[i\right]=\alpha_{1}+\beta_{1}\times male\left[i\right]+v_{1}\left[i\right],\;$$

and

(2) $$\lambda_{2}\left[i\right]=\alpha_{2}+\beta_{2}\times male\left[i\right]+v_{2}\left[i\right].$$

Here, the constants $\alpha_{1}$ and $\alpha_{2}$ represent log baseline hazards for stroke and CAD, respectively, and the coefficients $\beta_{1}$ and $\beta_{2}$ represent the corresponding log HR of male sex. The last terms $v_{1}$[*i*] and $v_{2}$[*i*] capture the unobserved heterogeneity in individual risks for stroke and CAD, respectively, which differ among individuals. Stroke and CAD are thromboembolic events that plausibly share an underlying pathophysiology. Indeed, they share the same predisposing factors, such as disease [4], lifestyle [27,28], socioeconomics [28], and genetics [29]. Accordingly, the susceptibility to stroke and CAD is likely to be positively correlated. Considering the correlation, we assumed that the unobserved individual risks $v_{1}$[*i*] and $v_{2}$[*i*] followed bivariate normal distribution with mean 0 and standard deviations (SD) $\sigma_{1}$ and $\sigma_{2}$ with a correlation ρ:

(3) $$\left(\begin{array}{c} v_{1}\\ v_{2} \end{array}\right)\sim N_{2}\left(0,\;\Sigma \right),\;\mathrm{where}\;\Sigma =\left(\begin{array}{cc} \sigma_{1}^{2} & \rho \sigma_{1}\sigma_{2}\\ \rho \sigma_{1}\sigma_{2} & \sigma_{2}^{2} \end{array}\right)$$

### 2.3. Parameters (Table 1)

We set the parameters such that the simulation generally reproduced real-world observations. We set the log HR $\beta_{2}$ of male sex for CAD to 0.7 or 1.2 such that the simulations reproduced the HR of male sex for CAD reported in the real world [18,19,20]. We assumed no effect of sex on AF-related stroke ($\beta_{1}$ = 0). However, we additionally evaluated the scenarios where male sex slightly increased stroke risk in AF ($\beta_{1}$ = 0.2). For individual heterogeneity terms, we examined 12 combinations of variability within populations and the correlation between the risks. For the variability, we presumed two levels ($\sigma_{1}$, $\sigma_{2}$ = 1.0 and 2.0) based on our previous simulation on the development of stroke in AF populations [34], in which the SD = 1.85 for individual risks reproduced the HR = 2.4 of prior stroke. For the correlations, we assumed three levels: high, moderate, and no correlation (ρ = 0.8, 0.4, and 0). The time $T_{e}$ for AF diagnosis was set arbitrarily because it was unit-free (we can scale it as desired). For the fixed $T_{e}$, we set the baseline hazard $\alpha_{2}$ = 0.2 of CAD such that it generated reasonable proportions of left-truncated persons. We set the baseline hazard $\alpha_{1}$ = 0.2 for stroke to the same value as CAD, such that the proportions of stroke among simulated AF patients largely agreed with the real-world observations [11]. In addition, to simulate various populations worldwide with a relative predominance of CAD to stroke, we examined a total of nine combinations of different baseline hazards for CAD and stroke ($\alpha_{1}$, $\alpha_{2}$ = 0.1, 0.5, and 1.0). The follow-up period $T_{c}$ was set to fit relatively into the time $T_{e}$ at AF diagnosis, such that the ratio $T_{c}/\;T_{e}$ was comparable to the ratio of the real-world study periods to the interval between CAD and AF onset. Finally, to assess the effect of informative censoring alone, we set the time of AF diagnosis $T_{e}$ = 0, which corresponds to young populations with early-onset AF.

**Table 1:** Parameters.

Symbol	Parameter	Value
$\alpha_{1}$	Log baseline hazard for stroke in AF	0.2
$\alpha_{2}$	Log baseline hazard for CAD	0.2
$\beta_{1}$	Log HR of male for stroke in AF	0, 0.2
$\beta_{2}$	Log HR of male for CAD	0.7, 1.2
$\sigma_{1}$	Standard deviation of stroke risks within populations	1.0, 2.0
$\sigma_{2}$	Standard deviation of CAD risks within populations	1.0, 2.0
ρ	Correlation of frailties between stroke and CAD	0, 0.4, 0.8
$T_{e}$	Time to AF diagnosis (arbitrary unit)	0.03
$T_{c}$	Period of follow-up after AF diagnosis	0.01

### 2.4. Simulation

We conducted 1000 simulations for each scenario. For each simulation, we generated a “population of AF” and conducted a “cohort study” to estimate the HR of female sex. Starting with an initial population of 80,000 individuals per sex, we first generated the time $T_{2}$ to CAD for each person. Of those who did not develop CAD before AF diagnosis at time $T_{e}$, we randomly registered 50,000 individuals per sex who constituted the “simulated cohort” of patients with AF. We subsequently conducted a “simulated cohort study” following the cohort over the $T_{c}$ period and recorded the time $T_{1}$ from registration to stroke for each person. When a person developed CAD before stroke during the follow-up, they were censored at the development of CAD. Finally, we estimated the HR by fitting a Cox proportional hazard model on the simulated cohort data consisting of the triplets $\left[\min\left(T_{1},\;T_{c},\;T_{2}-T_{e}\right),\;\delta ,\;\mathrm{sex}\right]$, where the indicator δ is 1 if stroke occurred and 0 if censored. The simulation was performed using R 4.2.0 (R Core Team, https://www.R-project.org/ accessed on 1 February 2023).

## 3. Results

Of the initial population, 75–89% of men and 88–94% of women were diagnosed with AF while alive (Supplementary Table S1). In all scenarios, a greater proportion of men were left-truncated than women, with a difference of 5–13%. The HR of male sex for CAD estimated in the initial populations ranged from 1.31 to 1.32 and from 1.62 to 1.67 for $\beta_{2}$ = 0.7 and 1.2, respectively. 

The HR of female sex exceeded one whenever a correlation existed between stroke and CAD risk (Table 2). Across the scenarios, there were some tendencies in the magnitude of HR. First, the higher the correlation between stroke and CAD risk, the higher the HR. Second, the wider the stroke and CAD risk distributions within the population, the higher the HR. Finally, the more susceptible men were to CAD than women, the higher the HR. Even when we assumed that male sex increased the risk of AF-related stroke, female sex was identified as a risk factor in some scenarios (Supplementary Table S2). 

In younger populations without LT, the HR of female sex was substantially lower than that of the corresponding scenarios in older populations (Table 3), underlining the importance of informative LT. Among populations with various combinations of CAD and stroke incidence, the HR was higher when CAD incidence was higher and when stroke incidence was lower (Table 4).

**Figure 3:**
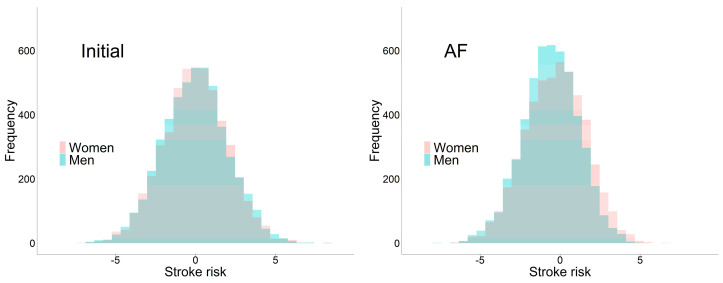
Distribution of stroke risk (log hazard) within initial (**left**) and AF (after LT, **right**) populations. Each histogram represents 5000 persons per sex randomly sampled from the corresponding population. Parameters were set to $\sigma_{1}$ = 2,$\;\sigma_{2}$ = 2, ρ = 0.8, $\beta_{1}$ = 0, and $\beta_{2}$ = 1.2.

From time 0 to AF diagnosis, the distribution of stroke risk shifted differently between sexes, such that high-risk men preferentially disappeared (Figure 3), illustrating the role of informative LT in generating the non-causal association.

## 4. Discussion

Our simulation demonstrates that a variable can be identified as a risk factor even if it does not cause the outcome of interest. This spurious association arises when the variable reduces the risk of a preceding CR that shares common unobserved causes with the outcome. We found that this association was generated mainly through informative LT by the correlated CR events. Importantly, the spurious association was not due to longevity alone because it arose only when the unobserved risks for CR and stroke were correlated. The results of this study suggest that some of the observed stroke predictions by female sex in AF may be due to men with a high stroke risk dropping out through correlated events, rather than women being inherently more prone to experiencing a stroke.

This phenomenon may be regarded as an instance of collider bias in a causal context: conditioning on a result (survival until AF diagnosis) creates an association between originally independent causes (sex and unobserved susceptibility) [35]. From this viewpoint, the differential shift in stroke risk between the sexes (Figure 3) can be interpreted in the following manner: “men in AF cohort, who, in spite of being male, have survived until AF diagnosis, tend to have lower unobserved susceptibility to stroke than women.” This bias occasionally presents an apparent paradox such as the “low birth-weight paradox” [36], but the bias may go unnoticed unless overtly paradoxical [35]. Researchers interested in causation should pay attention to potential bias, not necessarily a paradox, introduced by informative LT via preceding CR. 

We postulated that CAD is a major CR contributing to informative LT because in general (1) it occurs earlier than AF [2,37,38] and (2) associated mortality is higher and occurs earlier among men than among women [18,19,20,37,38,39]. Furthermore, it is plausible that the risks of CAD and stroke, both of which are thromboembolic events, are correlated through shared predisposing factors, including cardiometabolic diseases [4], lifestyle [27,28], socioeconomics [28], and genetics [29]. In addition to CAD and other cardiovascular events [18,38,40], CR could include any disease if mortality is higher and/or earlier in men and is correlated with stroke. Some diseases, such as cancer [30], hepatic diseases [31], and chronic obstructive pulmonary diseases [32,33] may satisfy this condition and therefore could act as a CR, contributing to informative LT [8,41]. 

The results of this study may explain some puzzling observations in AF populations regarding stroke risk prediction based on female sex. First, the dependency on age (female sex predicts stroke only in older populations) [5,6,7,8,9,10] can be explained by the different latencies during which CR events proceed (Table 2 as opposed to Table 3). Second, dependency on ethnicity (female sex predicts stroke in European populations but not in Asian populations) [5,11,12,13,14,15] can be explained by the different baseline hazards of CAD and stroke (Table 4). In summary, inconsistent risk prediction by female sex may have resulted from varying degrees of informative LT across populations. In addition, our hypothesis is consistent with another paradoxical observation in AF: stroke occurs more frequently in women, whereas CAD, cardiovascular mortality, and overall mortality occur more frequently in men [5,21]. Although the proposed mechanism is based solely on simulations, the potential explanation of several paradoxical observations suggests that our hypothesis warrants further investigation. 

It is important to note that our hypothesis does not contradict potential sex differences in the atrial substrate. Prior studies reported that a deteriorated atrial substrate in AF was associated with increased stroke risk and was more prevalent in women [42,43]. However, this female predominance became less pronounced or was even reversed in young patients [43,44], analogous to the “paradox” in stroke risk. Furthermore, greater deterioration in women is paradoxical given the higher incidence of AF in men [3]. Some sex differences in the atrial substrate may be generated by LT through CR. 

Although a risk factor is sometimes misinterpreted as causative, it is a predictor of disease irrespective of its causal role [45]. For example, prior stroke in AF is a non-causal, “Bayesian” risk factor upon which the presence of underlying causes is inferred from the result that the stroke occurred [34,46]. Female sex may be another non-causal risk factor—what one might call a “paradoxical” risk factor—upon which an apparent association is generated through informative LT. There may be such “paradoxical” factors in other diseases where the same structure exists. An example might be the female predominance in the incidence of Alzheimer’s disease [47]. 

This study had several limitations. First, our hypothesis remains to be verified in cohort studies, which may pose a significant challenge because left-truncated individuals are never observed. Additionally, several technical limitations were identified. First, our model has some simplifications, such as constant hazards and the homogenous development of AF. Second, because of the unobservable nature of LT, we started with a hypothetical population of “potential AF patients.” Because there is no real-world counterpart, we could not calibrate our LT process (development of CAD), although we believe that our model generated a reasonable LT considering the common predisposing factors between AF and CAD [3,48]. Third, we calibrated the hazard of CR only to CAD to maintain simplicity. Owing to these limitations, our estimations may not be quantitative. Finally, a caveat has been added. Although the phenomenon illustrated in this study may pose a challenge to causation, it does not pose any to prediction. Even if our hypothesis is correct, female sex could be a useful predictor of stroke in appropriate settings. 

## 5. Conclusions

We demonstrated that risk factors can be revealed in the absence of causal roles through left truncation due to preceding competing risks correlated with the outcome of interest. Female sex in AF may be a paradoxical risk factor for stroke among patients with AF.

## Figures and Tables

**Table 2:** HR of females for stroke in AF.

	Log HR of Male for CAD					
	$\beta_{2}=0.7$			$\beta_{2}=1.2$		
SD	Correlation			Correlation		
$\left(\sigma_{1},\;\sigma_{2}\right)$	ρ=0	ρ=0.4	ρ=0.8	ρ=0	ρ=0.4	ρ=0.8
**(1.0, 1.0)**	1.00 (0.046)	1.03 (0.049)	1.06 (0.049)	1.00 (0.044)	1.05 (0.048)	1.13 (0.054)
**(1.0, 2.0)**	1.00 (0.046)	1.04 (0.052)	1.10 (0.057)	1.00 (0.046)	1.08 (0.052)	1.20 (0.064)
**(2.0, 1.0)**	1.00 (0.026)	1.04 (0.029)	1.11 (0.031)	1.00 (0.026)	1.09 (0.032)	1.25 (0.036)
**(2.0, 2.0)**	1.00 (0.027)	1.08 (0.032)	1.22 (0.043)	1.00 (0.027)	1.15 (0.035)	1.45 (0.055)

Estimates (standard deviations) from 1000 simulations.

**Table 3:** HR of female sex for stroke in younger populations with AF (no left truncation).

	Log HR of Male for CAD					
	$\beta_{2}=0.7$			$\beta_{2}=1.2$		
SD	Correlation			Correlation		
$\left(\sigma_{1},\;\sigma_{2}\right)$	ρ=0	ρ=0.4	ρ=0.8	ρ=0	ρ=0.4	ρ=0.8
**(1.0, 1.0)**	1.00 (0.036)	1.01 (0.036)	1.01 (0.037)	1.00 (0.036)	1.01 (0.037)	1.02 (0.038)
**(1.0, 2.0)**	1.00 (0.037)	1.02 (0.038)	1.04 (0.040)	1.00 (0.037)	1.03 (0.039)	1.08 (0.042)
**(2.0, 1.0)**	1.00 (0.021)	1.01 (0.021)	1.02 (0.021)	1.00 (0.021)	1.01 (0.022)	1.03 (0.022)
**(2.0, 2.0)**	1.00 (0.021)	1.03 (0.022)	1.07 (0.025)	1.00 (0.022)	1.05 (0.023)	1.15 (0.027)

Estimates (standard deviations) from 1000 simulations.

**Table 4:** HR of female sex for stroke across populations of various baseline risk.

		Stroke Risk		
		Low	Moderate	High
**CAD risk**	**Low**	1.22 (0.045)	1.21 (0.035)	1.19 (0.029)
	**Moderate**	1.25 (0.051)	1.24 (0.042)	1.22 (0.034)
	**High**	1.29 (0.055)	1.27 (0.045)	1.26 (0.035)

Estimates (standard deviations) from 1000 simulations Low, moderate, and high risks correspond to the baseline hazards of 0.1, 0.5, and 1.0, respectively. Other parameters are set to $\sigma_{1}$ = 2,$\;\sigma_{2}$ = 2, ρ = 0.8, $\beta_{1}$ = 0, and $\beta_{2}$ = 0.7. The estimated HR of male sex for CAD in the initial populations was 1.31 in all scenarios.

## Data Availability

The code and parameters used for simulation are described in the Supplementary Materials and the article, respectively.

## Supplementary Materials

The following supporting information can be downloaded at https://www.mdpi.com/article/10.3390/life13051132/s1, Table S1: Proportion (%) of AF in initial populations and of stroke in AF populations; Table S2: HR of female when female sex is protective against stroke in AF; R code for simulation.
