# Supplementary material for: Female “Paradox” in Atrial Fibrillation—Role of Left Truncation Due to Competing Risks
Source: Life (Basel). 2023 May 5;13(5):1132. doi: 10.3390/life13051132 (PMC10220944; doi:10.3390/life13051132)
Supplement: Supplementary file 1 [file life-13-01132-s001.zip › Supplementary_tables.pdf]

Supplementary materials

Table S1. Proportion (%) of AF in initial populations and of stroke in AF populations

(σ_1, σ_2)	Population	$\beta_2 = 0.7$			$\beta_2 = 1.2$		
		$\rho = 0$	$\rho = 0.4$	$\rho = 0.8$	$\rho = 0$	$\rho = 0.4$	$\rho = 0.8$
(1.0, 1.0)	Entry(AF)/Initial, M	89	89	89	84	84	84
	Entry(AF)/Initial, F	94	94	94	94	94	94
	Stroke/AF, M	1.9	1.8	1.7	1.9	1.8	1.6
	Stroke/AF, F	1.9	1.9	1.8	1.9	1.9	1.8
(1.0, 2.0)	Entry(AF)/Initial, M	81	81	81	75	75	75
	Entry(AF)/Initial, F	88	88	88	88	88	88
	Stroke/AF, M	1.9	1.7	1.4	1.9	1.6	1.3
	Stroke/AF, F	1.9	1.8	1.5	1.9	1.8	1.5
(2.0, 1.0)	Entry(AF)/Initial, M	89	89	89	84	84	84
	Entry(AF)/Initial, F	94	94	94	94	94	94
	Stroke/AF, M	5.6	5.1	4.5	5.6	4.9	4.0
	Stroke/AF, F	5.6	5.4	5.0	5.6	5.4	5.0
(2.0, 2.0)	Entry(AF)/Initial, M	81	81	81	75	75	75
	Entry(AF)/Initial, F	88	88	88	88	88	88
	Stroke/AF, M	5.6	4.4	2.9	5.6	4.1	2.4
	Stroke/AF, F	5.6	4.8	3.5	5.6	4.8	3.5

Means from 1,000 simulations.

Table S2. HR of female when female sex is protective* against stroke in AF.

	Log HR of male for CAD					
	$\beta_2 = 0.7$			$\beta_2 = 1.2$		
SD	Correlation			Correlation		
(σ_1, σ_2)	$\rho = 0$	$\rho = 0.4$	$\rho = 0.8$	$\rho = 0$	$\rho = 0.4$	$\rho = 0.8$
(1.0, 1.0)	0.82	0.84	0.87	0.82	0.86	0.93
	(0.036)	(0.038)	(0.038)	(0.035)	(0.038)	(0.042)
(1.0, 2.0)	0.82	0.86	0.90	0.82	0.89	0.98
	(0.036)	(0.040)	(0.044)	(0.036)	(0.040)	(0.049)
(2.0, 1.0)	0.86	0.89	0.94	0.85	0.93	1.06
	(0.022)	(0.024)	(0.025)	(0.022)	(0.026)	(0.028)
(2.0, 2.0)	0.86	0.91	1.02	0.85	0.97	1.20
	(0.022)	(0.026)	(0.034)	(0.022)	(0.028)	(0.042)

Estimates (standard deviations) from 1,000 simulations. *Log HR β_1=0.2 of male for stroke
